# Hypoxia-triggered autophagy modulates cisplatin resistance in non-small cell lung Cancer via EIF2AK3-dependent PI3K/AKT signaling and mTOR-independent mechanisms

**DOI:** 10.1038/s41420-025-02893-z

**Published:** 2025-12-06

**Authors:** Jiding Fu, Wei Xu, Ge Wang, Lisi Zeng, Lewu Xian, Yier Wei, Jian Zhang

**Affiliations:** 1 Department of Intensive Care Unit, Affiliated Cancer Hospital & Institute of Guangzhou Medical University, Guangzhou, Guangdong China; 2 Thoracic Surgery, Affiliated Cancer Hospital & Institute of Guangzhou Medical University, Guangzhou, Guangdong China; 3 Radiation Oncology, Affiliated Cancer Hospital & Institute of Guangzhou Medical University, Guangzhou, Guangdong China; 4 Institute of Oncology, Affiliated Cancer Hospital & Institute of Guangzhou Medical University, Guangzhou, Guangdong China

**Keywords:** Non-small-cell lung cancer, Mechanisms of disease

## Abstract

Chemoresistance in non-small-cell lung cancer (NSCLC) remains a significant clinical challenge, often exacerbated by the tumor microenvironment’s hypoxic conditions. Hypoxia has been implicated in promoting autophagy and contributing to chemoresistance, yet the underlying mechanisms are not fully elucidated. In this study, we investigated the role of EIF2AK3 in hypoxia-induced autophagy and cisplatin (DDP) resistance in NSCLC cells. Our findings demonstrated that hypoxia upregulates EIF2AK3 expression, leading to enhanced autophagy, as indicated by increased LC3-II/I ratios. Pharmacological inhibition of autophagy with 3-MA effectively reversed hypoxia-induced DDP resistance. Mechanistically, hypoxia-activated EIF2AK3 enhanced autophagy and decreased DDP sensitivity in NSCLC cells via PI3K/AKT signaling, independent of mTOR activity. Activation of autophagy by rapamycin counteracted the effects of EIF2AK3 knockdown on both autophagy and PI3K/AKT signaling. Consistently, EIF2AK3 silencing in xenograft models enhanced the therapeutic efficacy of DDP by suppressing autophagy and attenuating PI3K/AKT activation. Collectively, our findings indicate that EIF2AK3 is a critical regulator of hypoxia-triggered autophagy in NSCLC, and targeting EIF2AK3-mediated PI3K/AKT signaling may represent a promising strategy to overcome cisplatin resistance.

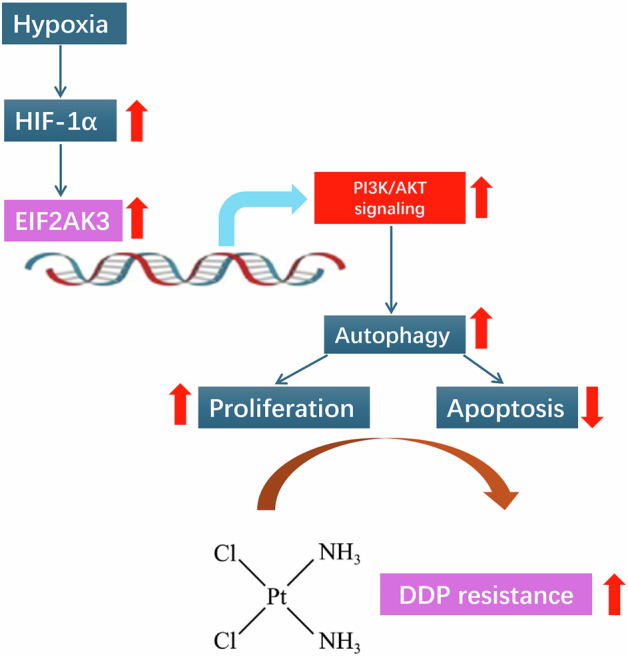

## Introduction

Non-small-cell lung cancer (NSCLC) stands as the most predominant subtype of malignant lung tumor, comprising approximately 80–85% of lung cancer cases [1]. In clinical settings, only a minor portion of NSCLC patients are diagnosed at an early stage, while the majority are in the advanced or even metastatic stage, with a 5-year survival rate of less than 15% [2]. For patients with advanced unresectable tumors, cisplatin (DDP) is one of the commonly employed chemotherapeutic agents for NSCLC [3]. However, the frequent occurrence of drug resistance often leads to adverse reactions and increased recurrence rates, significantly impacting its efficacy for NSCLC patients [4, 5]. Consequently, a more in-depth understanding of the drug resistance mechanisms can aid in the development of strategies to overcome chemoresistance.

Autophagy, a major intracellular degradation system that delivers cytoplasmic materials to the lysosome for degradation, plays a dual role in tumors [6, 7]. Initially, it acts as a tumor suppressor, but as tumors advance and face hostile environments, it supports tumor survival in harsh microenvironments [8]. Moreover, autophagy has been shown to function as a protective mechanism that not only promotes tumor proliferation but also confers resistance to DDP-based chemotherapy [9–11]. By activating autophagy, tumor cells can adapt to and survive under various adverse conditions, including hypoxia [12]. Hypoxia occurs in 90% of solid tumors due to insufficient oxygen supply from angiogenesis to rapidly growing tumor masses [13, 14]. During hypoxia, hypoxia-inducible factor 1-alpha (HIF-1α), the main transcriptional regulator, is frequently overexpressed in tumors and promotes tumor cell growth and survival by controlling both glycolysis and angiogenesis [15]. Recent studies have demonstrated the associations between hypoxia-related pathways and autophagy activation in cancers. For instance, Huang et al. [16] showed that HIF-1α can regulate autophagy and the PI3K/AKT/mTOR signaling pathway, affecting the biological behavior of ovarian cancer cells. Yang et al. [17] also reported that hypoxia-induced autophagy promotes gemcitabine resistance in human bladder cancer cells through HIF-1α activation. Additionally, Peng et al. [18] elucidated a mechanism in which the hypoxia/Egr-1/autophagy axis may be involved in drug resistance in hepatocellular carcinoma. Notably, hypoxia-induced autophagy has been implicated in mediating chemoresistance in NSCLC [19]. Nevertheless, the regulatory molecular mechanisms by which hypoxia-induced autophagy influences chemotherapy resistance in NSCLC cells remain incompletely understood.

EIF2AK3 (eukaryotic translation initiation factor 2 alpha kinase 3), also known as PERK (protein kinase R-like endoplasmic reticulum kinase), is a type I transmembrane endoplasmic reticulum (ER) receptor possessing a serine/threonine cytoplasmic domain [20]. Acting as a vital regulator of the unfolded protein response (UPR) in response to ER stress, EIF2AK3/PERK is capable of strengthening the autophagy signaling pathway [21] and facilitating the augmentation of chemoresistance [22]. Recent investigations have revealed that hypoxia can interfere with protein folding within the ER, and the ensuing ER stress triggers the activation of UPR pathways through the induction of EIF2AK3/PERK [23]. Several studies have suggested that the induction of the UPR promotes tumor cell survival and empowers solid tumors to deal with hypoxia and growth factor deprivation [24, 25]. Significantly, EIF2AK3/PERK has been recognized as one of the ER-resident sensors associated with the risk of lung cancer [26]. Based on these observations, we hypothesize that EIF2AK3/PERK plays a key role in regulating hypoxia-induced autophagy and chemoresistance in NSCLC.

To validate this hypothesis, our research focused on examining the influence of hypoxia on NSCLC cells treated with the chemotherapeutic agent DDP. We also analyzed the impacts of autophagy on hypoxia-induced DDP resistance in NSCLC cells. Moreover, we further verified the role of EIF2AK3 in regulating hypoxia-induced autophagy and the PI3K/AKT/mTOR signaling pathway in NSCLC cells both in vitro and in vivo. This study elucidates a mechanism in which the hypoxia/EIF2AK3/autophagy axis is implicated in DDP resistance in NSCLC.

## Results

### Hypoxia upregulated EIF2AK3, HIF-1α, and autophagy in NSCLC cells

**Fig. 1:**
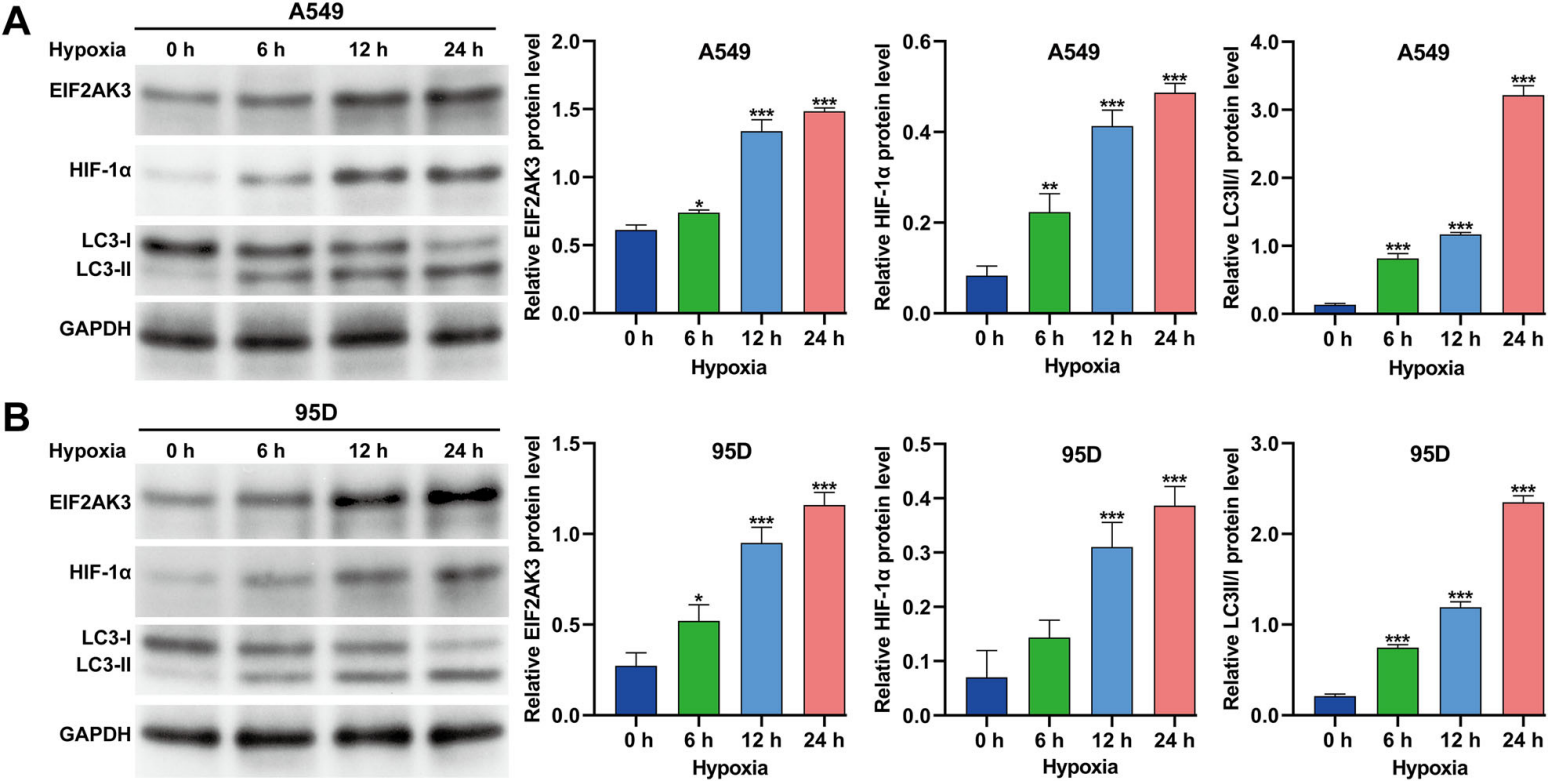
Hypoxia upregulated EIF2AK3, HIF-1α, and autophagy in NSCLC cells. A549 and 95D cells were exposed to hypoxia for different durations (0, 6, 12, and 24 h). Protein expression levels of EIF2AK3, HIF-1α, and the LC3-II/I ratio were measured, and corresponding quantitative analyses were performed for A549 (**A**) and 95D (**B**) cells. Data are presented as mean ± SD from three independent experiments. *p < 0.05, **p < 0.01, ***p < 0.001 versus 0 h.

To investigate the effects of hypoxia on EIF2AK3 expression and autophagy in NSCLC cells, A549 and 95D cells were incubated under hypoxic conditions for 0, 6, 12, and 24 h. Western blot analysis revealed that the protein levels of EIF2AK3, HIF-1α, and the LC3-II/I ratio gradually increased in a time-dependent manner in both cell lines (Fig. 1A, B). Quantitative analysis confirmed significant upregulation of these proteins under hypoxia. Based on these findings, 24 h of hypoxia was selected for subsequent experiments.

### Hypoxia-induced autophagy contributed to DDP resistance in NSCLC cells

**Fig. 2:**
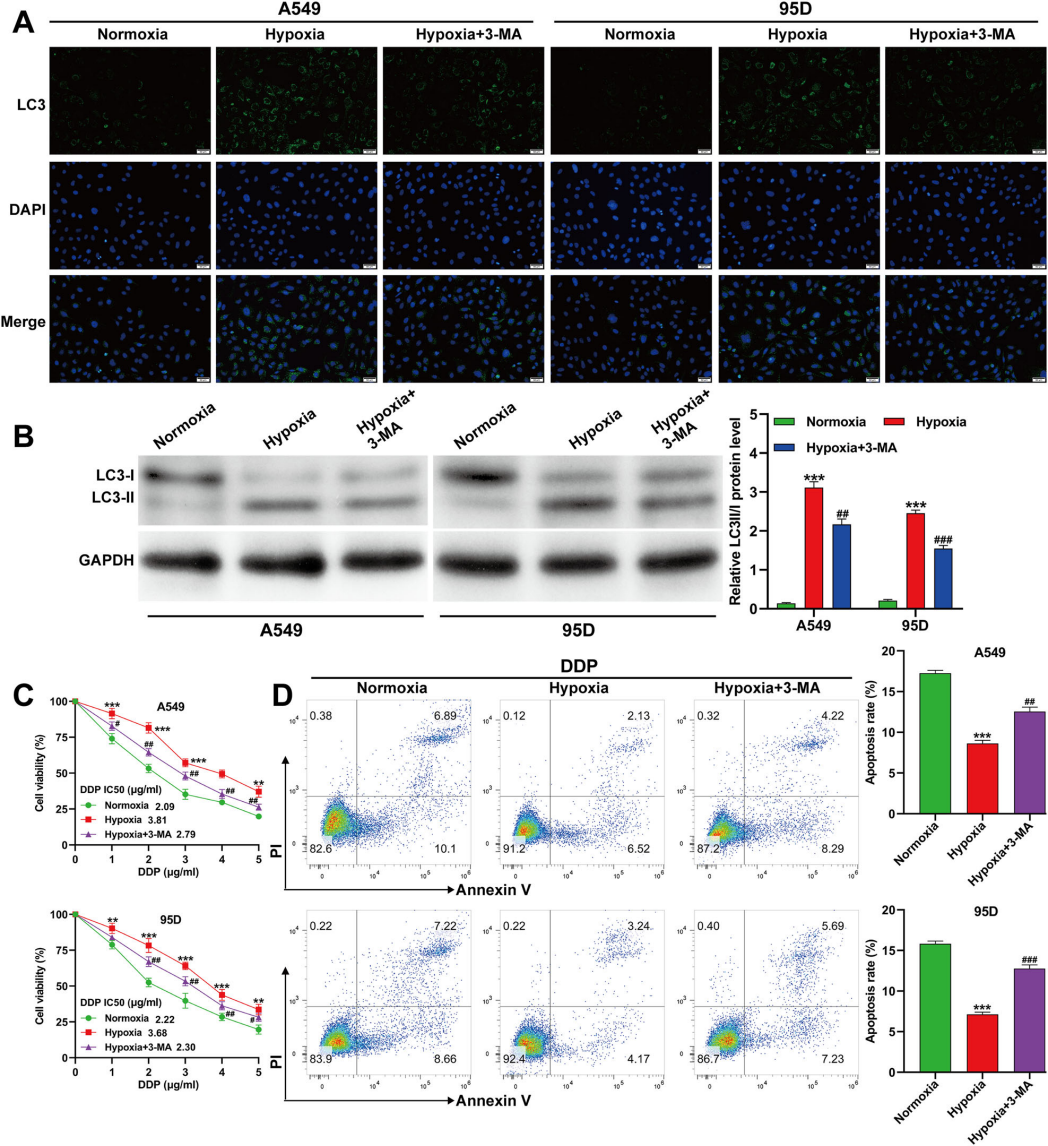
Hypoxia-induced autophagy contributed to DDP resistance in NSCLC cells. A549 and 95D cells were treated with 3-MA (10 mM) and then exposed to hypoxia for 24 h. **A** Representative immunofluorescence images of LC3 in A549 and 95D cells. **B** Protein expression of the LC3-II/I ratio and corresponding quantitative analyses in A549 and 95D cells. **C** Cell viability measured by CCK-8 assay in A549 and 95D cells treated with different concentrations of DDP under normoxia, hypoxia, or hypoxia + 3-MA for 24 h. **D** Apoptosis assessed by flow cytometry with Annexin V-FITC/PI staining in A549 and 95D cells treated with DDP (2 µg/ml) under normoxia, hypoxia, or hypoxia + 3-MA for 24 h. Data are presented as mean ± SD from three independent experiments. **p < 0.01, ***p < 0.001 versus normoxia; ##*p* < 0.01, ###*p* < 0.001 versus hypoxia.

To determine whether hypoxia-induced autophagy mediates DDP resistance, A549 and 95D cells were treated with the autophagy inhibitor 3-MA and exposed to hypoxia for 24 h. Immunofluorescence staining showed that the increased LC3 puncta observed under hypoxia were reversed by 3-MA treatment (Fig. 2A). Western blot analysis similarly demonstrated that the elevated LC3-II/I ratio under hypoxia was attenuated by 3-MA (Fig. 2B). CCK-8 assays revealed that 3-MA reversed hypoxia-induced increases in cell viability after DDP treatment in a dose-dependent manner (Fig. 2C). Flow cytometry confirmed that hypoxia reduced DDP-induced apoptosis, which was restored by 3-MA (Fig. 2D). These results indicate that autophagy plays a critical role in hypoxia-mediated DDP resistance in NSCLC cells.

### EIF2AK3 knockdown suppressed autophagy and enhanced DDP cytotoxicity in NSCLC cells

**Fig. 3:**
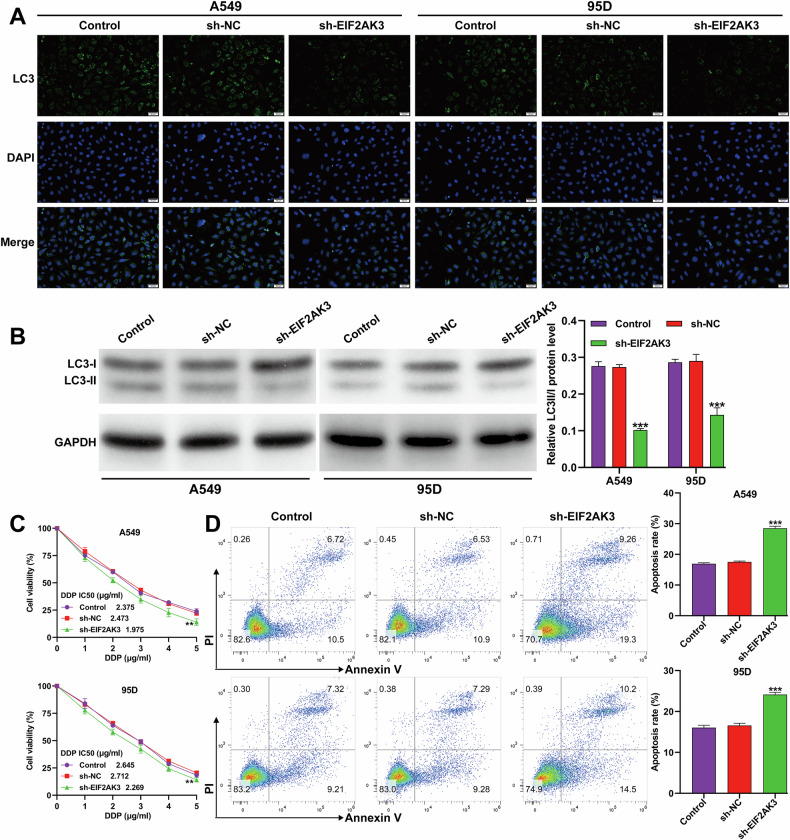
EIF2AK3 knockdown suppressed autophagy and enhanced DDP cytotoxicity in NSCLC cells. A549 and 95D cells were transduced with sh-EIF2KA3 or sh-NC for 48 h. **A** Representative immunofluorescence staining images of LC3 in transduced A549 and 95D cells. **B** Protein expression of the LC3-II/I ratio and corresponding quantitative analyses in transduced A549 and 95D cells. **C** Cell viability was detected by CCK-8 assay in transduced A549 and 95D cells cultured with different doses (0, 1, 2, 3, 4, and 5 μg/ml) of DDP. **D** Cell apoptosis was detected by flow cytometry assay with Annexin-V FITC/PI double staining in transduced A549 and 95D cells. Data are presented as mean ± SD from three independent experiments. **p < 0.01, ***p < 0.001 versus sh-NC.

Loss-of-function assays were conducted to explore the role of EIF2AK3 in autophagy and DDP resistance. Immunofluorescence and western blot analyses demonstrated that LC3 expression was markedly reduced in A549 and 95D cells transduced with sh-EIF2AK3 (Fig. 3A, B). Under normoxia, EIF2AK3 knockdown decreased cell viability in response to DDP in a dose-dependent manner (Fig. 3C) and significantly increased apoptosis (Fig. 3D), indicating that EIF2AK3 contributes to basal autophagy and chemoresistance.

### EIF2AK3 enhanced hypoxia-induced autophagy and DDP resistance in NSCLC cells

**Fig. 4:**
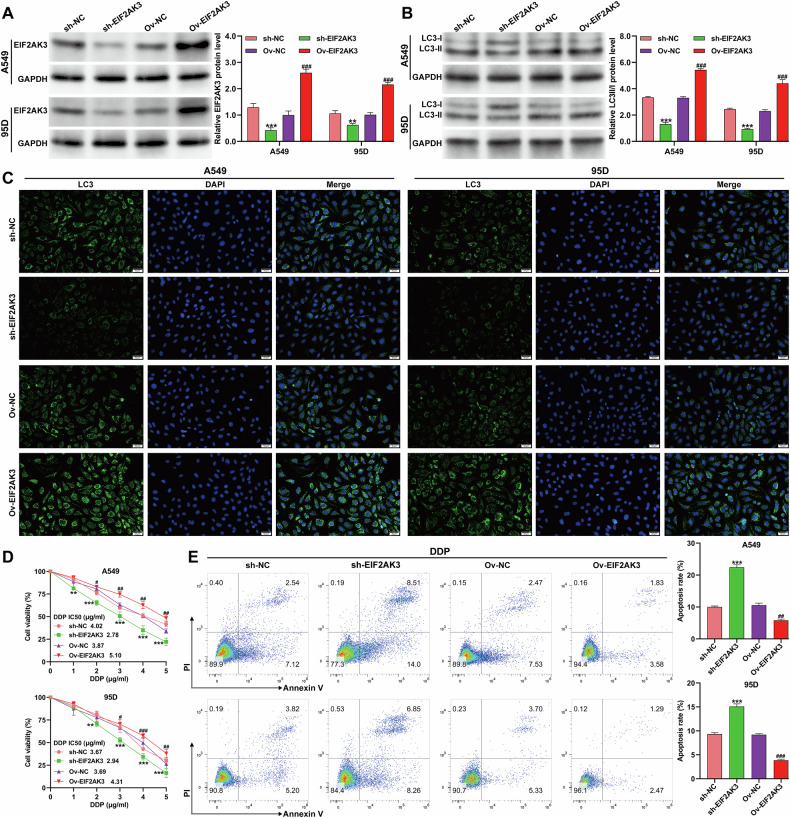
EIF2AK3 enhanced hypoxia-induced autophagy and DDP resistance in NSCLC cells. A549 and 95D cells were transduced with sh-EIF2KA3 or Ov-EIF2AK3 and then exposed to hypoxia for 24 h. Western blot analysis was carried out to determine the protein expression of EIF2AK3 (**A**) and the ratio of LC3-II/I (**B**) in the above A549 and 95D cells. **C** Representative immunofluorescence staining images of LC3 in the above-mentioned A549 and 95D cells. **D** Cell viability was assessed by CCK-8 assay in the above A549 and 95D cells cultured with various doses (0, 1, 2, 3, 4, and 5 μg/ml) of DDP. **E** Cell apoptosis was detected by flow cytometry assay using Annexin-V FITC/PI double staining in the above A549 and 95D cells exposed to DDP (2 µg/ml) under hypoxia. Data are presented as mean ± SD from three independent experiments. **p < 0.01, ***p < 0.001 versus sh-NC; #*p* < 0.05, ##*p* < 0.01, ###*p* < 0.001 versus Ov-NC.

To assess the role of EIF2AK3 in hypoxia-induced DDP resistance, A549 and 95D cells were transduced with sh-EIF2AK3 or Ov-EIF2AK3 and exposed to hypoxia. Under hypoxic conditions, Western blot analysis confirmed effective knockdown of EIF2AK3 in A549 and 95D cells transduced with sh-EIF2AK3, and successful overexpression of EIF2AK3 in cells transduced with Ov-EIF2AK3 (Fig. 4A). Correspondingly, the levels of the autophagy marker LC3-II/I were decreased following EIF2AK3 knockdown and increased upon EIF2AK3 overexpression in both cell lines (Fig. 4B). These changes were further validated by immunofluorescence staining of LC3 (Fig. 4C). Functional assays revealed that EIF2AK3 knockdown enhanced DDP cytotoxicity and apoptosis under hypoxia, whereas overexpression conferred resistance (Fig. 4D, E). These results indicate that EIF2AK3 is a key mediator of hypoxia-induced autophagy and DDP resistance.

### EIF2AK3 modulated PI3K/AKT signaling independently of mTOR in NSCLC cells

**Fig. 5:**
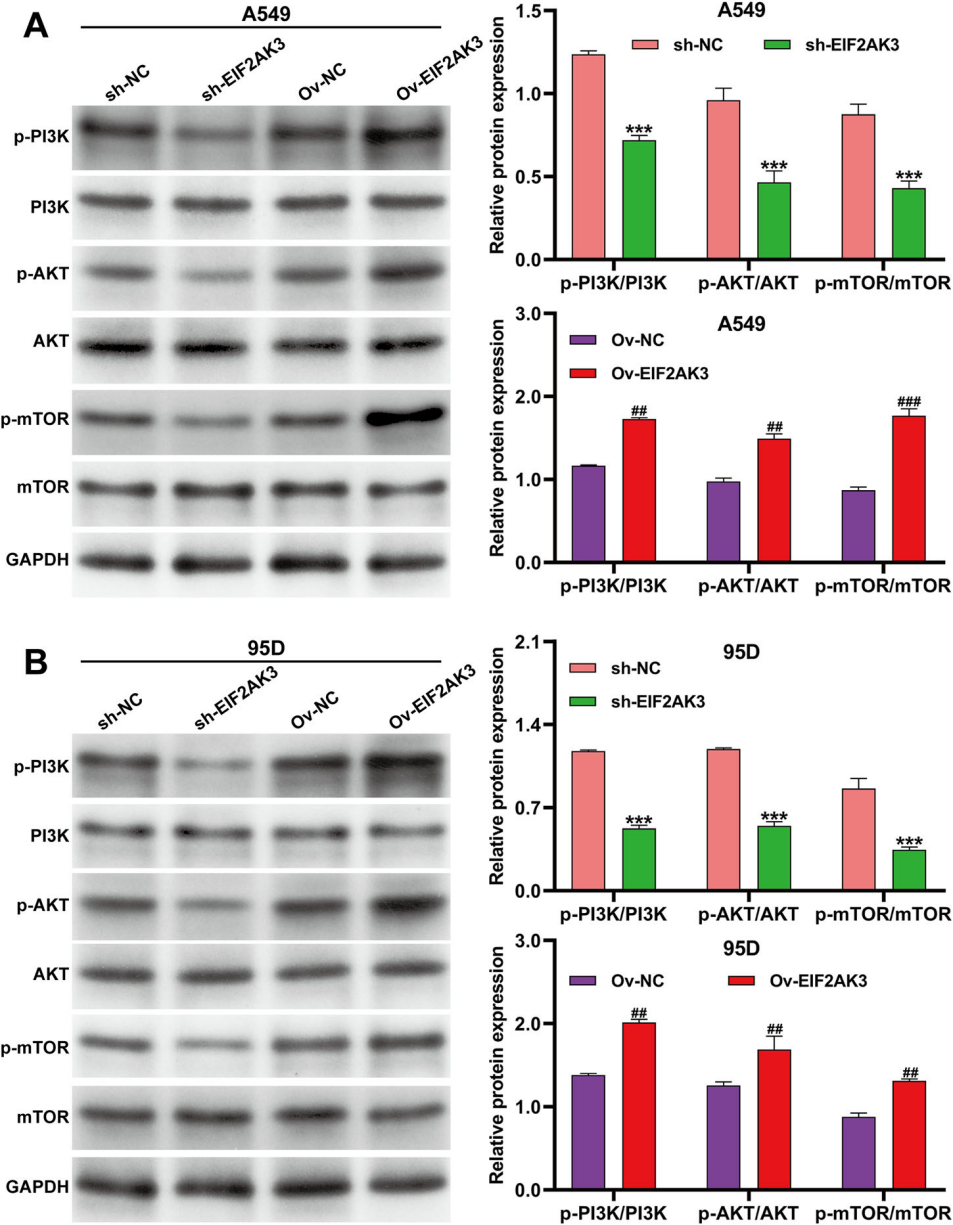
EIF2AK3 modulated PI3K/AKT signaling independently of mTOR in NSCLC cells. A549 and 95D cells were transduced with sh-EIF2KA3 or Ov-EIF2AK3 and then exposed to hypoxia for 24 h. Western blot analysis was performed to assess the protein expression of PI3K, p-PI3K, AKT, p-AKT, mTOR, and p-mTOR in A549 (**A**) and 95D (**B**) cells. Data are presented as mean ± SD from three independent experiments. *p < 0.05, **p < 0.01, ***p < 0.001 versus sh-NC; #*p* < 0.05, ##*p* < 0.01 versus Ov-NC.

The PI3K/AKT signaling pathway is a critical regulator of cell survival and drug resistance in cancer. To investigate whether EIF2AK3 influences this pathway under hypoxia, we examined the expression of PI3K, AKT, and mTOR proteins in A549 and 95D cells. Knockdown of EIF2AK3 significantly decreased the ratios of p-PI3K/PI3K and p-AKT/AKT, whereas overexpression of EIF2AK3 markedly increased these phosphorylation levels in both cell lines (Fig. 5A, B). Notably, the changes in p-mTOR/mTOR were minimal and inconsistent, suggesting that EIF2AK3 primarily modulates hypoxia-induced DDP resistance through PI3K/AKT activation, independently of canonical mTOR signaling. These findings indicate that EIF2AK3 promotes chemoresistance in NSCLC cells by activating the PI3K/AKT pathway, while mTOR may not be the main downstream effector in this context.

### Autophagy activation reversed effects of EIF2AK3 knockdown in NSCLC cells

**Fig. 6:**
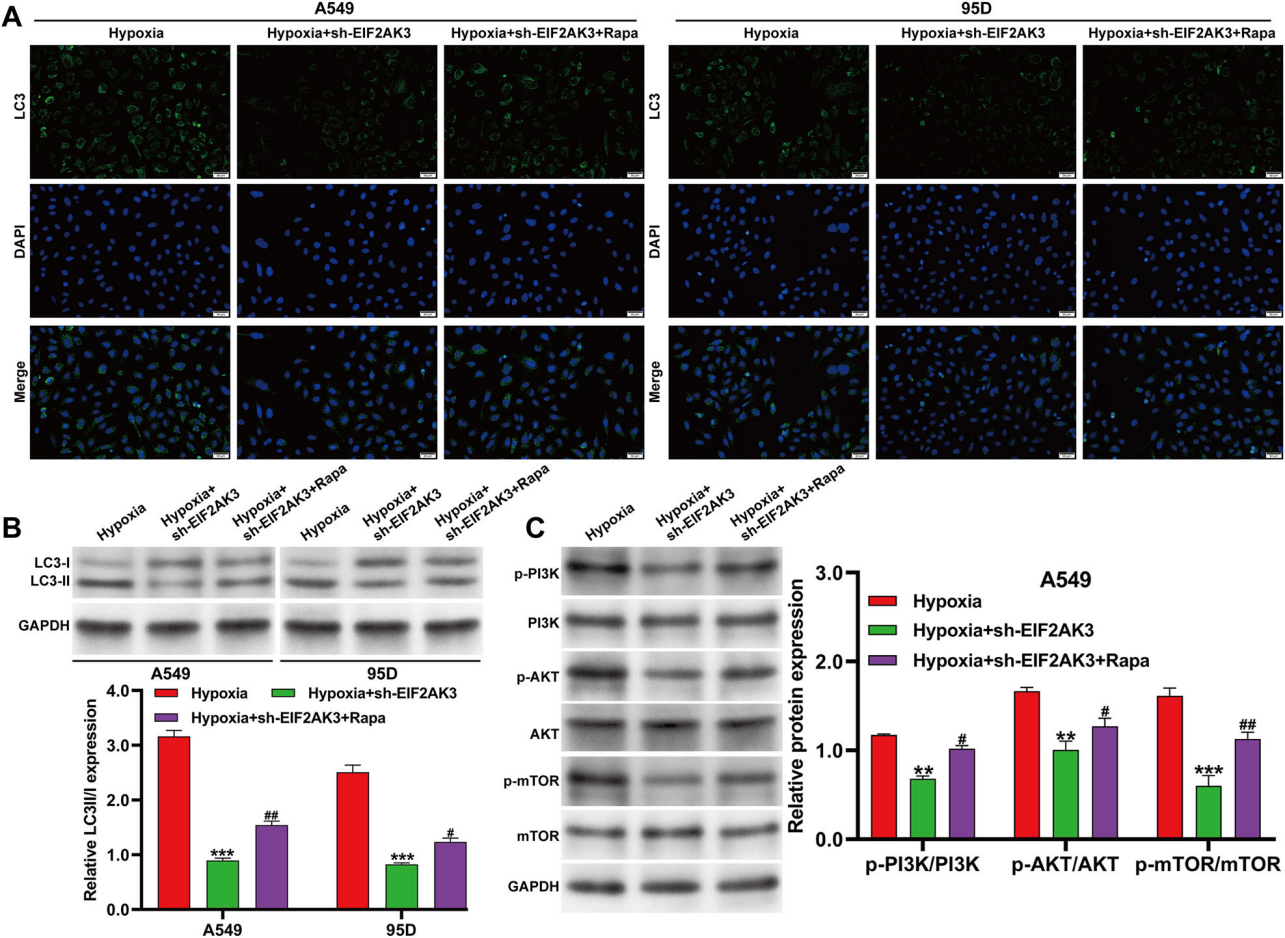
Autophagy activation reversed effects of EIF2AK3 knockdown in NSCLC cells. A549 and 95D cells transduced with sh-EIF2AK3 were treated with Rapa (10 nM, an autophagy activator) and then exposed to hypoxia. **A** Representative immunofluorescence images of LC3 in the treated A549 and 95D cells. **B** Western blot analysis of the LC3-II/I ratio in these cells. **C** Western blot analysis of PI3K, p-PI3K, AKT, p-AKT, mTOR, and p-mTOR expression in A549 cells. Data are presented as mean ± SD from three independent experiments. **p < 0.01, ***p < 0.001 versus hypoxia; #*p* < 0.05, ##*p* < 0.01 versus hypoxia + sh-EIF2AK3.

To confirm the contribution of autophagy, we treated A549 and 95D cells transduced with sh-EIF2AK3 with rapamycin (Rapa, 10 μM), a known autophagy activator, followed by hypoxia exposure. Immunofluorescence staining showed that Rapa treatment reversed the decreased LC3-positive puncta caused by EIF2AK3 knockdown in both cell lines (Fig. 6A). Western blot analysis further demonstrated that the reduced LC3-II/LC3-I ratio observed after EIF2AK3 knockdown was significantly restored by Rapa treatment (Fig. 6B). Consistent with these results, rapamycin treatment reversed the suppressive effects of sh-EIF2AK3 on p-PI3K and p-AKT levels. Notably, p-mTOR levels were also partially restored by Rapa under these conditions (Fig. 6C). Given that Rapa classically inhibits mTOR activity, this partial restoration of p-mTOR likely reflects complex regulation of mTOR phosphorylation, including possible feedback or compensatory mechanisms in hypoxia-exposed NSCLC cells. Therefore, p-mTOR levels should be interpreted with caution, and further investigation is required to understand the mechanism.

### EIF2AK3 knockdown enhanced DDP efficacy in vivo via autophagy inhibition

**Fig. 7:**
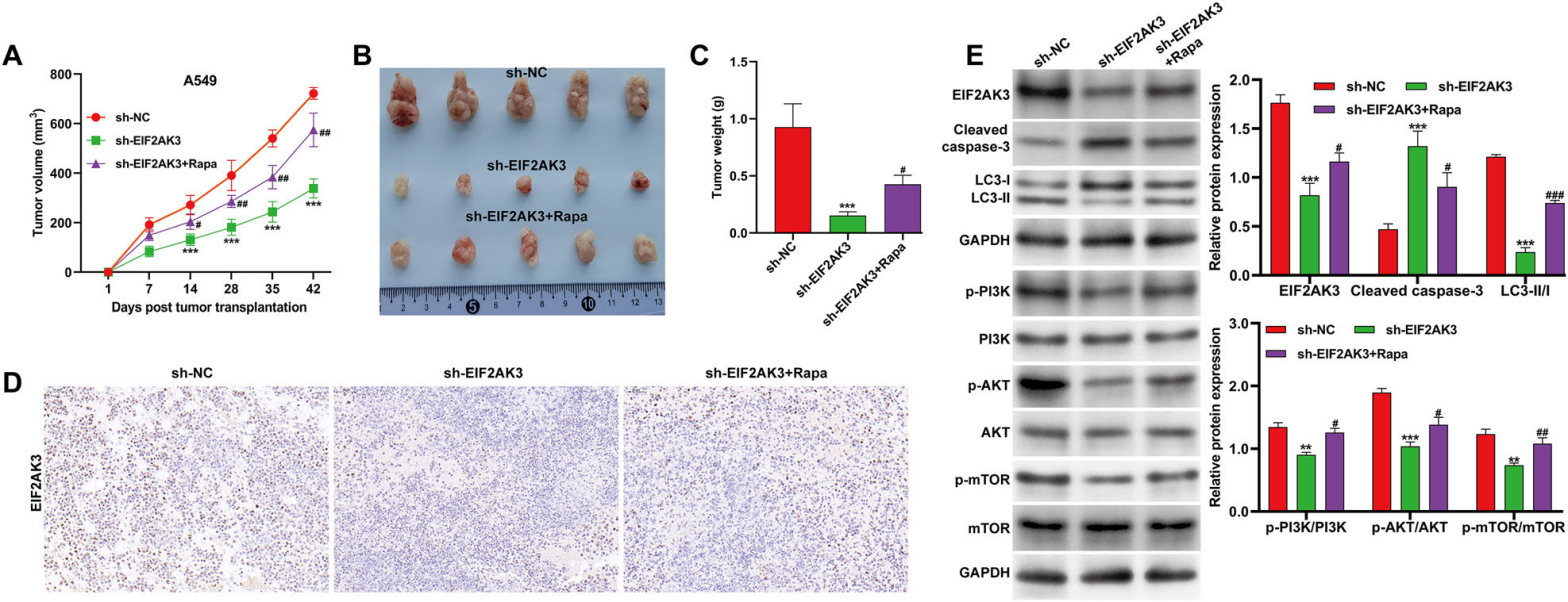
EIF2AK3 knockdown enhanced DDP efficacy in vivo via autophagy inhibition. BALB/c nude mice were inoculated with A549 cells (5 × 10^6^) stably transduced with sh-EIF2AK3, or sh-NC (*n* = 5 per group). Once the average tumor volume reached 50 mm³, mice were administered intraperitoneal injections of DDP (20 mg/kg daily for 7 days) and/or rapamycin (Rapa, 2 mg/kg every other day for 2 weeks), with PBS serving as the control. (**A**) Tumor volume was continuously monitored for 42 days. On day 42, the xenografts were removed (**B**) and weighed (**C**). **D** The protein expression of EIF2AK3 in xenograft tumor tissues was determined by immunohistochemistry assay. **E** The protein levels of EIF2AK3 cleaved caspase-3, LC3-II/I ratio, PI3K, p-PI3K, AKT, p-AKT, mTOR, and p-mTOR in xenograft tumor tissues were measured. Data are presented as mean ± SD from three independent experiments (*n* = 5 per group). **p < 0.01, ***p < 0.001 versus sh-NC; #*p* < 0.05, ##*p* < 0.01, ###*p* < 0.001 versus sh-EIF2AK3.

To investigate the role of EIF2AK3 in DDP resistance in vivo, a xenograft model was established using A549 cells transduced with sh-EIF2AK3 or sh-NC. When the average tumor volume reached approximately 50 mm^3^, mice received intraperitoneal injections of DDP and/or Rapa. As shown in Fig. 7A, EIF2AK3 knockdown significantly reduced tumor growth, an effect that was partially reversed by Rapa treatment. By day 42, both the size (Fig. 7B) and weight (Fig. 7C) of tumors derived from sh-EIF2AK3-transduced A549 cells were markedly lower compared with control tumors after DDP treatment, and Rapa co-treatment attenuated this reduction. Immunohistochemistry confirmed efficient knockdown of EIF2AK3 in tumor tissues, which was mitigated by Rapa (Fig. 7D). Western blot analysis demonstrated increased cleaved caspase-3 and decreased levels of LC3-II/I, p-PI3K, p-AKT, and p-mTOR in sh-EIF2AK3 tumors, all of which were reversed by Rapa (Fig. 7E). Notably, while EIF2AK3 knockdown consistently decreased p-mTOR in both in vitro and in vivo conditions, autophagy activation with Rapa led to partial restoration of p-mTOR levels, indicating a complex feedback regulation during autophagy induction. These findings suggest that EIF2AK3 mediates DDP resistance primarily via autophagy-dependent PI3K/AKT signaling, with mTOR phosphorylation being contextually modulated.

## Discussion

Despite recent advances in improving the efficacy of DDP in NSCLC treatment, the development of drug resistance remains a major challenge, often resulting in therapeutic failure, tumor progression, or recurrence [27]. Autophagy has been increasingly recognized as a cytoprotective mechanism that shields cancer cells from chemotherapy-induced apoptosis [28]. Among the stimuli that induce autophagy, hypoxia is a critical factor that enables cancer cells to survive under chemotherapeutic stress [29]. In our study, we observed that hypoxia exposure led to time-dependent increases in HIF-1α expression and the LC3-II/I ratio in A549 and 95D cells. Inhibition of autophagy by 3-MA effectively abolished hypoxia-induced DDP resistance. These findings align with clinical observations that hypoxia is prevalent in NSCLC and can activate multiple pro-survival pathways [30]. HIF-1α, induced under hypoxic conditions, is known to promote autophagy and chemotherapy resistance across diverse cancer types [31, 32]. Taken together, our data suggest that targeting hypoxia-driven autophagy may represent a viable approach to overcome DDP resistance in NSCLC therapy.

Components of the unfolded protein response (UPR) are frequently overexpressed in cancer, and UPR-related factors are emerging as potential therapeutic targets [33]. EIF2AK3 (PERK), a central UPR regulator, has been implicated in chemoresistance across multiple cancer types [34]. In our study, EIF2AK3 expression was markedly induced by hypoxia in NSCLC cells in a time-dependent manner. Our in vitro experiments demonstrated that EIF2AK3 knockdown enhanced DDP cytotoxicity under normoxic conditions and attenuated hypoxia-induced autophagy and DDP resistance. In vivo, knockdown of EIF2AK3 in A549 xenografts resulted in smaller tumors and enhanced responsiveness to DDP chemotherapy. ER stress, caused by protein misfolding in the secretory pathway, profoundly impacts cancer cell survival [35]. EIF2AK3-mediated ER stress has been linked to increased autophagy in tumor tissues, such as in colorectal cancer [36]. Consistently, inhibition of PERK/eIF2α signaling enhances chemotherapeutic responses by promoting apoptosis in small-cell lung cancer [37] and chronic myeloid leukemia models [38]. These findings support a signaling cascade whereby hypoxia induces HIF-1α, which upregulates EIF2AK3, subsequently triggering autophagy to protect NSCLC cells from DDP-induced cytotoxicity.

In light of the as-yet-undetermined mechanism, we focused on the PI3K/AKT signaling pathway, a well-established route associated with autophagy regulation in cancer cells [39]. PI3K phosphorylates AKT, which in turn modulates multiple downstream targets that govern cell survival, proliferation, and apoptosis [40]. Although mTOR, an atypical serine/threonine kinase, participates in sensing extracellular signals such as nutrients, energy, and growth factors and regulates processes including protein synthesis, ribosome biogenesis, and programmed cell death [41, 42], our results indicate that EIF2AK3 primarily regulates autophagy through PI3K/AKT signaling independently of mTOR. Overexpression of EIF2AK3 enhanced autophagic flux, as evidenced by increased LC3-II/I ratio, concomitant with PI3K/AKT activation, whereas mTOR phosphorylation did not consistently correlate with autophagic changes. This appears paradoxical because canonical mTOR activation inhibits autophagy mainly through phosphorylation of ULK1 at Ser757. Although ULK1 Ser757 phosphorylation was not assessed in this study, previous reports have demonstrated that EIF2AK3 (PERK) can induce autophagy via mTOR-independent mechanisms. This occurs through the PERK–eIF2α–ATF4 signaling axis, which transcriptionally upregulates autophagy-related genes (e.g., ATG5, BECN1, LC3) and can modulate AMPK activity, thereby promoting autophagy despite active mTOR signaling [43, 44]. In our rescue experiments, rapamycin treatment not only restored autophagy markers but also partially reversed the suppression of p-mTOR induced by EIF2AK3 knockdown under hypoxia. This observation appears contradictory to the classical understanding that rapamycin inhibits mTOR activity to induce autophagy. However, phosphorylation levels of mTOR detected by Western blot may not fully reflect its kinase activity or functional status, as different phosphorylation sites exert distinct regulatory effects. Moreover, under hypoxic conditions and EIF2AK3 deficiency, complex feedback loops or compensatory signaling may cause transient or context-dependent changes in p-mTOR levels. Therefore, caution should be exercised when interpreting p-mTOR levels as sole indicators of autophagy regulation. Further mechanistic studies are needed to elucidate the dynamic interplay between EIF2AK3, autophagy, and the PI3K/AKT/mTOR signaling pathway in NSCLC cells.

In conclusion, our study demonstrates that hypoxia-induced autophagy is a critical mechanism driving DDP resistance in NSCLC cells. EIF2AK3 mediates this response primarily through activation of the PI3K/AKT signaling pathway, independently of mTOR, both in vitro and in vivo. While mTOR phosphorylation exhibits complex and context-dependent regulation under hypoxia, the PI3K/AKT axis remains a consistent downstream effector of EIF2AK3 in promoting autophagy and chemoresistance. Targeting EIF2AK3 and its PI3K/AKT-mediated autophagic pathway represents a promising strategy for overcoming DDP resistance and improving therapeutic outcomes in NSCLC patients.

## Materials and methods

### Cell culture and treatment

Human NSCLC cell lines (A549 and 95D) were procured from the Cell Bank of the Chinese Academy of Sciences (Shanghai, China). The identity of both cell lines was authenticated by short tandem repeat (STR) profiling, and the cells were routinely tested for mycoplasma contamination using PCR-based methods. These cells were cultured in DMEM medium supplemented with 100 μg/ml streptomycin, 10% FBS, and 100 U/ml penicillin (Gibco, Grand Island, NY, USA). Normoxic conditions were maintained in a humidified incubator at 37 °C with 21% O_2_ and 5% CO_2_. To induce hypoxia, cells were exposed for 0, 6, 12, or 24 h in a sealed chamber containing a gas mixture of 1% O_2_, 5% CO_2_, and 94% N_2_. Under hypoxic conditions, cells were treated with the autophagy activator rapamycin (Rapa, 100 nM, 12 h) or the inhibitor 3-methyladenine (3-MA, 5 mM, 2 h) (Sigma-Aldrich) to examine the role of autophagy in hypoxia-induced resistance.

### Cell transfection

The short hairpin RNA against EIF2AK3 (sh-EIF2AK3) and a negative control shRNA (sh-NC) were designed and cloned into the GV248 lentiviral vector (GenePharma Co., Ltd., Shanghai, China). For EIF2AK3 overexpression (Ov-EIF2AK3), the full-length human EIF2AK3 open reading frame (ORF) was amplified by PCR and cloned into the GV341 lentiviral expression vector (Shanghai Genechem). An empty vector was used as a negative control (Ov-NC). Recombinant lentiviruses were generated by co-transfecting the lentiviral constructs with packaging plasmids into HEK293T cells using Lipofectamine 2000 (Life Technologies). After 48 h of transfection, the viral supernatants were collected and used to infect A549 and 95D cells in the presence of 6 μg/mL polybrene (Sigma-Aldrich) for 48 h. Stably transduced cells were then selected with 2 μg/mL puromycin in fresh culture medium.

### Drug sensitivity assay

Drug sensitivity was assessed using the Cell Counting Kit-8 (CCK-8) assay (Dojindo Laboratories, Tokyo, Japan) following the manufacturer’s instructions. Briefly, NSCLC cells subjected to various treatments were seeded in triplicate into 96-well plates at a density of 1 × 10^4^ cells per well. Cells were then treated with different concentrations of DDP (0, 1, 2, 3, 4, and 5 μg/ml; Sigma-Aldrich) for 24 h. After treatment, 20 μl of CCK-8 reagent was added to each well, and the cells were incubated for 2 h at 37 °C. The optical density (OD) at 450 nm was measured using a microplate reader (Bio-Rad Laboratories), and the relative cell survival rate was calculated using the formula: Cell survival rate (%) = (OD _experimental_ – OD _blank_)/(OD _control_ – OD _blank_) × 100%. The IC50 values of DDP for NSCLC cells were determined from dose-response curves with variable slopes. All experiments were performed independently in triplicate.

### Apoptosis analysis

The proportion of NSCLC cells undergoing apoptosis was assessed by flow cytometry. After 24 h of treatment with DDP (2 µg/ml), NSCLC cells subjected to different treatments were collected, washed once with PBS, and resuspended at a final concentration of 1 × 10^6^ cells/ml. Cells were then stained in the dark at room temperature for 15 min using a binding buffer containing Annexin V-fluorescein isothiocyanate (FITC) and propidium iodide (PI) (KeyGEN BioTECH, Nanjing, China). Apoptosis was subsequently analyzed using a FACS flow cytometry (Becton Dickinson, Franklin Lakes, NJ, USA). All experiments were independently performed in triplicate.

### Immunofluorescence assay

NSCLC cells subjected to various treatments and cultured in flat-bottomed dishes were fixed with 4% paraformaldehyde, followed by permeabilization with 0.5% Triton ×-100 for 10 min. After blocking with 5% bovine serum albumin (BSA), cells were incubated overnight at 4 °C with anti-LC3 primary antibody (ab232940; Abcam), followed by incubation with a fluorescein-conjugated secondary antibody. Nuclei were counterstained with DAPI (1 μg/ml). The immunofluorescent images were captured using a confocal laser scanning microscope (Leica, Wetzlar, Germany).

### Animal experiments

Considering sex was not considered as a biological variable, female BALB/c mice (4 weeks old, weighing 15–20 × *g*) were obtained from Beijing Vital River Laboratory Animal Technology Co., Ltd. (Charles River Laboratories, Wilmington, MA, USA). These mice were maintained under sterile conditions (40%–60% relative humidity, 26 °C–28 °C, and 12 h light/12 h dark cycle) with ad libitum access to food and water. Approximately 5 × 10^6^ A549 cells transduced with sh-EIF2AK3 or sh-NC were implanted into the right dorsal region near the forelimb of BALB/c nude mice. Once the average tumor volume reached 50 mm³, mice were administered intraperitoneal injections of DDP (20 mg/kg daily for 7 days) and/or rapamycin (Rapa, 2 mg/kg every other day for 2 weeks), with PBS serving as the control. All mice were randomly assigned to three groups (sh-NC, sh-EIF2AK3, and sh-EIF2AK3 + Rapa), with five mice per group. Randomization was implemented to minimize bias in group allocation. Investigators performing tumor measurements and data analysis were blinded to the group assignments. Tumor length (L) and width (W) were measured every 7 days using a sliding caliper, and tumor volume was calculated as (L × W^2^)/2. After 42 days, the mice were euthanized, and the xenografts were excised, weighed, and stored for further analysis. All animal procedures were conducted in strict accordance with the International Guiding Principles for Animal Research and were approved by the Animal Ethics Committee of the Affiliated Cancer Hospital & Institute of Guangzhou Medical University (Approval No. GMU-87AD, Guangdong, China).

### Immunohistochemistry (IHC)

Xenograft tumor tissues were fixed in 4% paraformaldehyde and sectioned into 4 μm-thick slices. The sections were then deparaffinized and rehydrated, followed by treatment with H₂O₂ to block endogenous peroxidase activity. After blocking with 5% BSA, the sections were incubated overnight at 4 °C with a primary antibody against EIF2AK3 (1:500, ab79483, Abcam), and subsequently with a secondary antibody for 2 h. The sections were stained with 3, 3′-diaminobenzidine (DAB) for 3 min, washed in water for 10 min, and counterstained with hematoxylin. Finally, the stained sections were observed and photographed at 200× magnification using a digitalized microscope (Nikon, Tokyo, Japan).

### Western blot analysis

Total protein was extracted using RIPA lysis buffer (Beyotime, Shanghai, China), and concentrations were determined with a BCA protein assay kit (Beyotime). Equal amounts of protein (30 µg) were separated on 10–12% SDS-PAGE gels and transferred to PVDF membranes (Millipore, USA). The membranes were blocked with 5% non-fat dry milk and incubated overnight at 4 °C with the following primary antibodies: EIF2AK3 (1:500, #AF5304, Affinity Biosciences), HIF-1α (1:400, ab1, Abcam), LC3 (1:10000, A27200PM, ABclonal), cleaved caspase-3 (1:1000, #9661, Cell Signaling), p-PI3K (1:1000, #17366, Cell Signaling), PI3K (1:1000, #4292, Cell Signaling), p-AKT (1:2000, A18675, ABclonal), AKT (1:500, AP0637, ABclonal), p-mTOR (1:1000, #55250, Cell Signaling), mTOR (1:1000, #2972, Cell Signaling) and GAPDH (1:50000, A19056, ABclonal). Membranes were then incubated with horseradish peroxidase (HRP)-conjugated secondary antibodies (1:5000, #7074, Cell Signaling, Danvers, MA, USA) for 1 h. Protein signals were detected using enhanced chemiluminescence reagents (Thermo Fisher Scientific, MA, USA), and band intensities were quantified with ImageJ software (NIH, Bethesda, MD, USA).

### Statistical analysis

Data were obtained from three independent experiments for each group, and results are presented as the mean ± standard deviation (SD). Comparisons between two groups were performed using Student’s *t*-test, while multiple-group comparisons were conducted using one-way analysis of variance (ANOVA) followed by Tukey’s post hoc test (GraphPad Prism 8.0). A *p*-value < 0.05 was considered statistically significant.

## Supplementary information

Original WB

## Data Availability

The datasets used and/or analyzed during the current study are available from the corresponding author on reasonable request.

## References

[1] Yang T, Xiong Y, Zeng Y, Wang Y, Zeng J, Liu J, et al. Current status of immunotherapy for non-small cell lung cancer. Front Pharm. 2022;13:989461. 10.3389/fphar.2022.989461 PMC9606217 36313314

[2] Mithoowani H, Febbraro M. Non-small-cell lung cancer in 2022: a review for general practitioners in oncology. Curr Oncol. 2022;29:1828–39. 35323350 10.3390/curroncol29030150 PMC8946954

[3] Song Y, Wang L, Zheng Y, Jia L, Li C, Chao K, et al. Deubiquitinating enzyme USP28 inhibitor AZ1 alone and in combination with cisplatin for the treatment of non-small cell lung cancer. Apoptosis. 2024;29:1793–809. 39222275 10.1007/s10495-024-02008-6 PMC11416398

[4] Kryczka J, Kryczka J, Czarnecka-Chrebelska KH, Brzezianska-Lasota E. Molecular mechanisms of chemoresistance induced by cisplatin in NSCLC cancer therapy. Int J Mol Sci. 2021;22:8885. 34445588 10.3390/ijms22168885 PMC8396273

[5] Chen H, Li F, Xue Q. Circ-CUL2/microRNA-888-5p/RB1CC1 axis participates in cisplatin resistance in NSCLC via repressing cell advancement. Bioengineered. 2022;13:2828–40. 35068326 10.1080/21655979.2021.2024395 PMC8974128

[6] Mizushima N, Komatsu M. Autophagy: renovation of cells and tissues. Cell. 2011;147:728–41. 22078875 10.1016/j.cell.2011.10.026

[7] Yu H, Zhuang J, Zhou Z, Song Q, Lv J, Yang X, et al. METTL16 suppressed the proliferation and cisplatin-chemoresistance of bladder cancer by degrading PMEPA1 mRNA in a m6A manner through autophagy pathway. Int J Biol Sci. 2024;20:1471–91. 38385084 10.7150/ijbs.86719 PMC10878153

[8] Poillet-Perez L, White E. Role of tumor and host autophagy in cancer metabolism. Genes Dev. 2019;33:610–9. 31160394 10.1101/gad.325514.119 PMC6546058

[9] He Z, Cai K, Zeng Z, Lei S, Cao W, Li X. Autophagy-associated circRNA circATG7 facilitates autophagy and promotes pancreatic cancer progression. Cell Death Dis. 2022;13:233. 35288538 10.1038/s41419-022-04677-0 PMC8921308

[10] Pan X, Chen Y, Shen Y, Tantai J. Knockdown of TRIM65 inhibits autophagy and cisplatin resistance in A549/DDP cells by regulating miR-138-5p/ATG7. Cell Death Dis. 2019;10:429. 31160576 10.1038/s41419-019-1660-8 PMC6546683

[11] Peng L, Sang H, Wei S, Li Y, Jin D, Zhu X, et al. circCUL2 regulates gastric cancer malignant transformation and cisplatin resistance by modulating autophagy activation via miR-142-3p/ROCK2. Mol Cancer. 2020;19:156. 33153478 10.1186/s12943-020-01270-x PMC7643398

[12] Mao X, Nanzhang, Xiao J, Wu H, Ding K. Hypoxia-induced autophagy enhances cisplatin resistance in human bladder cancer cells by targeting hypoxia-inducible factor-1alpha. J Immunol Res. 2021;2021:8887437. 33681390 10.1155/2021/8887437 PMC7904373

[13] Zhou J, Schmid T, Schnitzer S, Brune B. Tumor hypoxia and cancer progression. Cancer Lett. 2006;237:10–21. 16002209 10.1016/j.canlet.2005.05.028

[14] Cosse JP, Michiels C. Tumour hypoxia affects the responsiveness of cancer cells to chemotherapy and promotes cancer progression. Anticancer Agents Med Chem. 2008;8:790–7. 18855580 10.2174/187152008785914798

[15] Lee JG, Shin JH, Shim HS, Lee CY, Kim DJ, Kim YS, et al. Autophagy contributes to the chemo-resistance of non-small cell lung cancer in hypoxic conditions. Respir Res. 2015;16:138. 26553068 10.1186/s12931-015-0285-4 PMC4640373

[16] Huang J, Gao L, Li B, Liu C, Hong S, Min J, et al. Knockdown of hypoxia-inducible factor 1alpha (HIF-1alpha) promotes autophagy and inhibits phosphatidylinositol 3-kinase (PI3K)/AKT/mammalian target of rapamycin (mTOR) signaling pathway in ovarian cancer cells. Med Sci Monit. 2019;25:4250–63. 31175269 10.12659/MSM.915730 PMC6573092

[17] Yang X, Yin H, Zhang Y, Li X, Tong H, Zeng Y, et al. Hypoxia-induced autophagy promotes gemcitabine resistance in human bladder cancer cells through hypoxia-inducible factor 1α activation. Int J Oncol. 2018;53:215–24. 29693166 10.3892/ijo.2018.4376

[18] Peng WX, Xiong EM, Ge L, Wan YY, Zhang CL, Du FY, et al. Egr-1 promotes hypoxia-induced autophagy to enhance chemo-resistance of hepatocellular carcinoma cells. Exp Cell Res. 2016;340:62–70. 26708617 10.1016/j.yexcr.2015.12.006

[19] Wu HM, Jiang ZF, Ding PS, Shao LJ, Liu RY. Hypoxia-induced autophagy mediates cisplatin resistance in lung cancer cells. Sci Rep. 2015;5:12291. 26201611 10.1038/srep12291 PMC4511870

[20] Cui W, Li J, Ron D, Sha B. The structure of the PERK kinase domain suggests the mechanism for its activation. Acta Crystallogr D Biol Crystallogr. 2011;67:423–8. 21543844 10.1107/S0907444911006445 PMC3087621

[21] Kouroku Y, Fujita E, Tanida I, Ueno T, Isoai A, Kumagai H, et al. ER stress (PERK/eIF2alpha phosphorylation) mediates the polyglutamine-induced LC3 conversion, an essential step for autophagy formation. Cell Death Differ. 2007;14:230–9. 16794605 10.1038/sj.cdd.4401984

[22] Madden E, Logue SE, Healy SJ, Manie S, Samali A. The role of the unfolded protein response in cancer progression: from oncogenesis to chemoresistance. Biol Cell. 2019;111:1–17. 30302777 10.1111/boc.201800050

[23] Rozpedek W, Pytel D, Mucha B, Leszczynska H, Diehl JA, Majsterek I. The role of the PERK/eIF2α/ATF4/CHOP signaling pathway in tumor progression during endoplasmic reticulum stress. Curr Mol Med. 2016;16:533–44. 27211800 10.2174/1566524016666160523143937 PMC5008685

[24] Verfaillie T, Salazar M, Velasco G, Agostinis P. Linking ER stress to autophagy: potential implications for cancer therapy. Int J Cell Biol. 2010;2010:930509. 20145727 10.1155/2010/930509 PMC2817393

[25] Wang G, Yang ZQ, Zhang K. Endoplasmic reticulum stress response in cancer: molecular mechanism and therapeutic potential. Am J Transl Res. 2010;2:65–74. 20182583 PMC2826823

[26] Liu Y, Liang X, Zhang H, Dong J, Zhang Y, Wang J, et al. ER stress-related genes EIF2AK3, HSPA5, and DDIT3 polymorphisms are associated with risk of lung cancer. Front Genet. 2022;13:938787. 35923704 10.3389/fgene.2022.938787 PMC9341132

[27] Kildey K, Gandhi NS, Sahin KB, Shah ET, Boittier E, Duijf PHG, et al. Elevating CDCA3 levels in non-small cell lung cancer enhances sensitivity to platinum-based chemotherapy. Commun Biol. 2021;4:638. 34050247 10.1038/s42003-021-02136-8 PMC8163776

[28] Notte A, Ninane N, Arnould T, Michiels C. Hypoxia counteracts taxol-induced apoptosis in MDA-MB-231 breast cancer cells: role of autophagy and JNK activation. Cell Death Dis. 2013;4:e638. 23681233 10.1038/cddis.2013.167 PMC3674374

[29] Mazure NM, Pouyssegur J. Hypoxia-induced autophagy: cell death or cell survival?. Curr Opin Cell Biol. 2010;22:177–80. 20022734 10.1016/j.ceb.2009.11.015

[30] Xu G, Chen H, Wu S, Chen J, Zhang S, Shao G, et al. Eukaryotic initiation factor 5A2 mediates hypoxia-induced autophagy and cisplatin resistance. Cell Death Dis. 2022;13:683. 35931669 10.1038/s41419-022-05033-y PMC9356061

[31] Long F, Liu W, Jia P, Wang H, Jiang G, Wang T. HIF-1α-induced autophagy contributes to cisplatin resistance in ovarian cancer cells. Pharmazie. 2018;73:533–6. 30223937 10.1691/ph.2018.8514

[32] Mao X, Nanzhang, Xiao J, Wu H, Ding K. Hypoxia-induced autophagy enhances cisplatin resistance in human bladder cancer cells by targeting hypoxia-inducible factor-1α. J Immunol Res. 2021;2021:8887437. 33681390 10.1155/2021/8887437 PMC7904373

[33] Nagelkerke A, Bussink J, Sweep FC, Span PN. The unfolded protein response as a target for cancer therapy. Biochim Biophys Acta. 2014;1846:277–84. 25069067 10.1016/j.bbcan.2014.07.006

[34] Stockwell SR, Platt G, Barrie SE, Zoumpoulidou G, Te Poele RH, Aherne GW, et al. Mechanism-based screen for G1/S checkpoint activators identifies a selective activator of EIF2AK3/PERK signalling. PLoS ONE. 2012;7:e28568. 22253692 10.1371/journal.pone.0028568 PMC3257223

[35] Clarke HJ, Chambers JE, Liniker E, Marciniak SJ. Endoplasmic reticulum stress in malignancy. Cancer Cell. 2014;25:563–73. 24823636 10.1016/j.ccr.2014.03.015

[36] Jiang X, Li G, Zhu B, Yang J, Cui S, Jiang R, et al. p20BAP31 Induces autophagy in colorectal cancer cells by promoting PERK-mediated ER stress. Int J Mol Sci. 2024;25:5101. 38791141 10.3390/ijms25105101 PMC11121724

[37] Xu L, Jiang Y, Bi Y, Zheng S, Wu Y, Wu Y, et al. Suppression of PERK/eIF2alpha/CHOP pathway enhances oridonin-induced apoptosis by inhibiting autophagy in small-cell lung cancer cells. Biomed Pharmacother. 2024;175:116684. 38713951 10.1016/j.biopha.2024.116684

[38] Kusio-Kobialka M, Podszywalow-Bartnicka P, Peidis P, Glodkowska-Mrowka E, Wolanin K, Leszak G, et al. The PERK-eIF2α phosphorylation arm is a pro-survival pathway of BCR-ABL signaling and confers resistance to imatinib treatment in chronic myeloid leukemia cells. Cell Cycle. 2012;11:4069–78. 23095523 10.4161/cc.22387 PMC3507502

[39] He W, Cheng Y. Inhibition of miR-20 promotes proliferation and autophagy in articular chondrocytes by PI3K/AKT/mTOR signaling pathway. Biomed Pharmacother. 2018;97:607–15. 29101804 10.1016/j.biopha.2017.10.152

[40] Wang S, Zhu M, Wang Q, Hou Y, Li L, Weng H, et al. Alpha-fetoprotein inhibits autophagy to promote malignant behaviour in hepatocellular carcinoma cells by activating PI3K/AKT/mTOR signalling. Cell Death Dis. 2018;9:1027. 30301886 10.1038/s41419-018-1036-5 PMC6177398

[41] Kim YC, Guan KL. mTOR: a pharmacologic target for autophagy regulation. J Clin Invest. 2015;125:25–32. 25654547 10.1172/JCI73939 PMC4382265

[42] Husseinzadeh N, Husseinzadeh HD. mTOR inhibitors and their clinical application in cervical, endometrial and ovarian cancers: a critical review. Gynecol Oncol. 2014;133:375–81. 24556063 10.1016/j.ygyno.2014.02.017

[43] Rashid HO, Yadav RK, Kim HR, Chae HJ. ER stress: autophagy induction, inhibition and selection. Autophagy. 2015;11:1956–77. 26389781 10.1080/15548627.2015.1091141 PMC4824587

[44] Liu ZW, Zhu HT, Chen KL, Dong X, Wei J, Qiu C, et al. Protein kinase RNA-like endoplasmic reticulum kinase (PERK) signaling pathway plays a major role in reactive oxygen species (ROS)-mediated endoplasmic reticulum stress-induced apoptosis in diabetic cardiomyopathy. Cardiovasc Diabetol. 2013;12:158. 24180212 10.1186/1475-2840-12-158 PMC4176998

