# Supplementary figures and images for: Hypoxia-triggered autophagy modulates cisplatin resistance in non-small cell lung Cancer via EIF2AK3-dependent PI3K/AKT signaling and mTOR-independent mechanisms
Source: Cell Death Discov. 2025 Dec 6;12:40. doi: 10.1038/s41420-025-02893-z (PMC12827477; doi:10.1038/s41420-025-02893-z)

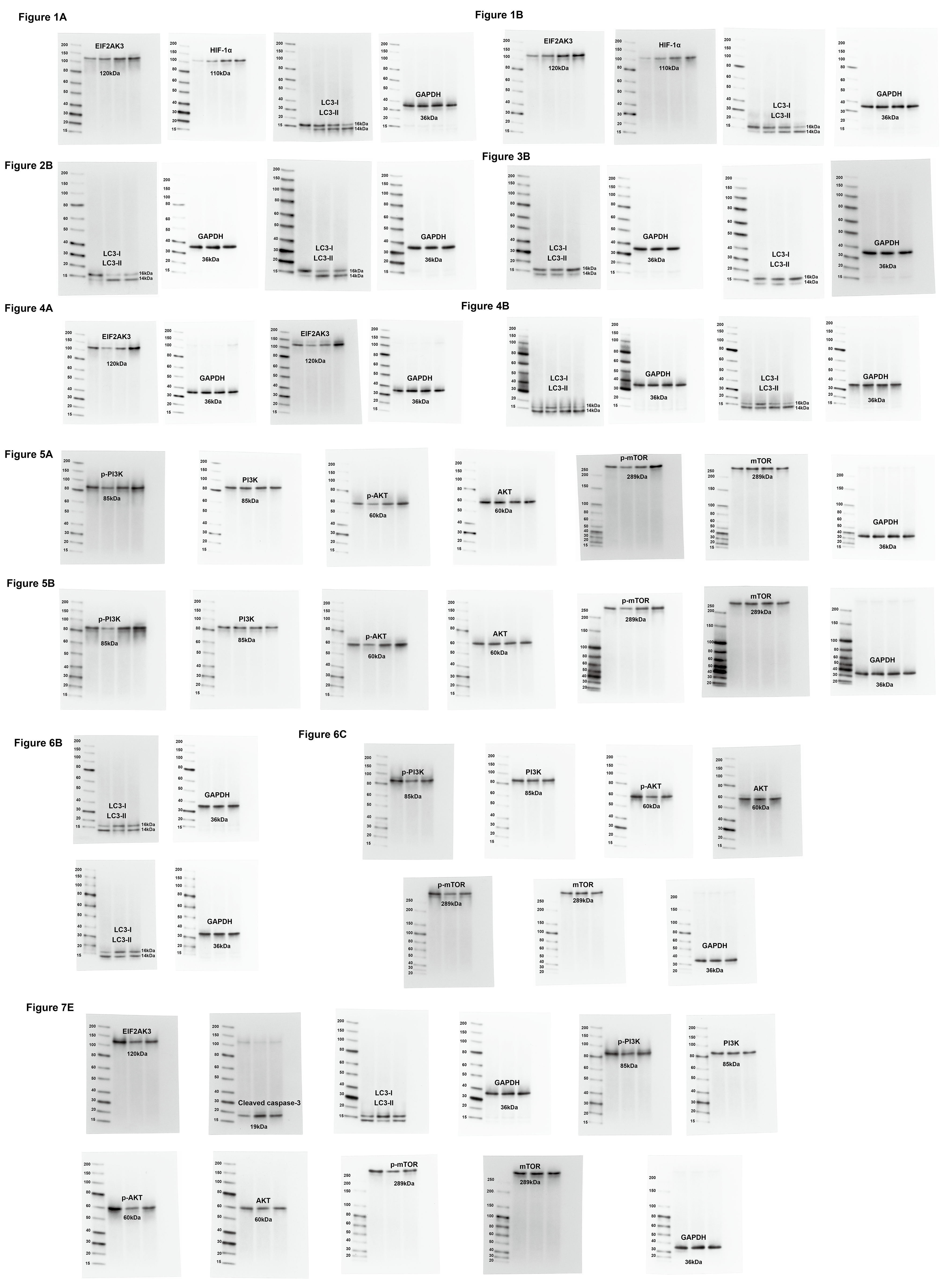

Supplement: Supplementary file 1 — Original WB [file 41420_2025_2893_MOESM1_ESM.tif]
